# Subphrenic abscess due to *Clostridium perfringens* after hepatic resection for hepatocellular carcinoma following emphysematous cholecystitis: Report of a case

**DOI:** 10.1016/j.ijscr.2020.01.029

**Published:** 2020-01-27

**Authors:** Ryoga Hamura, Koichiro Haruki, Yu Kumagai, Hiroaki Shiba, Shigeki Wakiyama, Katsuhiko Yanaga

**Affiliations:** Department of Surgery, The Jikei University School of Medicine, Tokyo, Japan

**Keywords:** CT, Computed tomography, HCC, Hepatocellular carcinoma, PTGBD, Percutaneous transhepatic gallbladder drainage, MRI, Magnetic resonance imaging, FMOX, Flomoxef Sodium, IPM/CS, Imipenem/Cilastatin, CPFX, Ciprofloxacin, CLDM, Clindamycin, DRPM, Doripenem, TAZ/PIPC, Tazobactam/Piperacillin hydrate, FRPM, Faropenem, Hepatic resection, Subphrenic abscess, *Clostridium perfringens*

## Abstract

**Background:**

Subphrenic abscess caused by *Clostridium perfringens* is rare after hepatic resection. We herein report such a case after hepatic resection for hepatocellular carcinoma following treatment of emphysematous cholecystitis.

**Case presentation:**

A 69-years-old man with chronic hepatitis B, was admitted to our hospital for right subcostal pain and loss of appetite. Computed tomography (CT) revealed emphysematous cholecystitis, for which percutaneous transhepatic gallbladder drainage was performed. *Clostridium perfringens* was identified from the culture of the bile. Imaging studies immediately demonstrated hepatocellular carcinoma with right lobe of the liver, for which the patients underwent hepatic resection and cholecystectomy concomitantly. After operation, the patient developed emphysematous subphrenic abscess on postoperative day 15, for which CT-guided percutaneous drainage was performed. *Clostridium perfringens* was identified from the culture of the abscess fluid. The patient was given Ciprofloxacin and Clindamycin and made a satisfactory recovery. The patient was discharged on POD 95 and remains well with no evidence of tumor recurrence as of 8 years after resection.

**Conclusion:**

We herein reported a subphrenic abscess due to *Clostridium perfringens* after hepatic resection for hepatocellular carcinoma following emphysematous cholecystitis.

## Highlights

• Postoperative subphrenic abscess caused by *Clostridium perfringens* is rare after hepatic resection.
• *Clostridium perfringens* causes severe sepsis with high mortality rate.
• Early antibiotic and surgical treatment for the focus of infection is significantly improve in the survival rate.

## 1 Background

Subphrenic abscess is one of the common complications after hepatic resection.　 *Clostridium perfringens* causes severe sepsis with high mortality rate, and only the resection or drainage of the infected lesion improve the outcome [1]. We herein report a case of subphrenic abscess due to *Clostridium perfringens* after concomitant hepatic resection and cholecystectomy for emphysematous cholecystitis and incidentally found hepatocellular carcinoma.

This paper has been reported in line with the SCARE criteria [2].

## 2 Case presentation

**Fig. 1:**
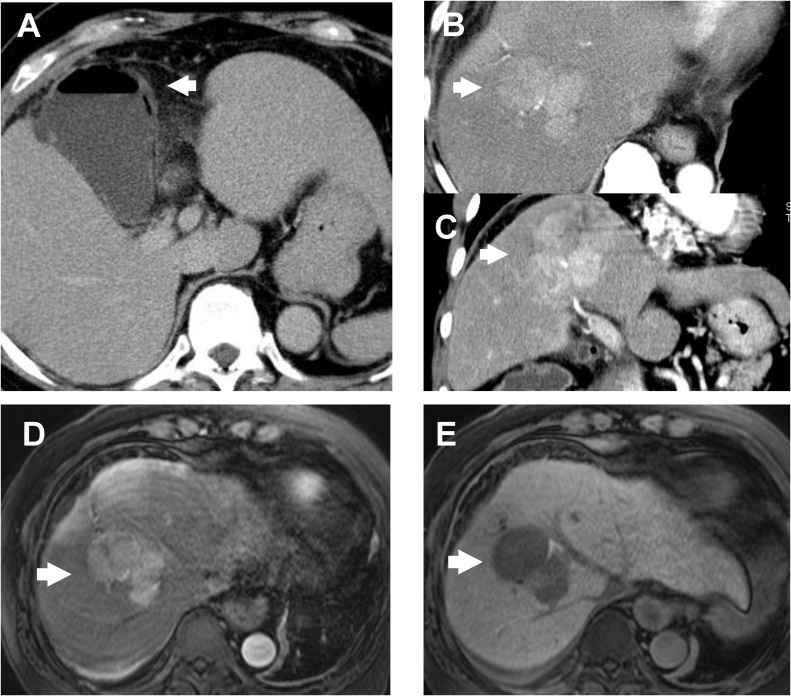
Abdominal computed tomography revealed wall thickness of the gallbladder with air density (A) and an 80-mm tumor with early enhancement in the segment 8 of the liver tumor (B–C, arrow). Gadoxetic acid-enhanced magnetic resonance imaging demonstrated a liver tumor, which had hyper vascularity in arterial phase (D) and washout in hepatocyte phase (E).

**Fig. 2:**
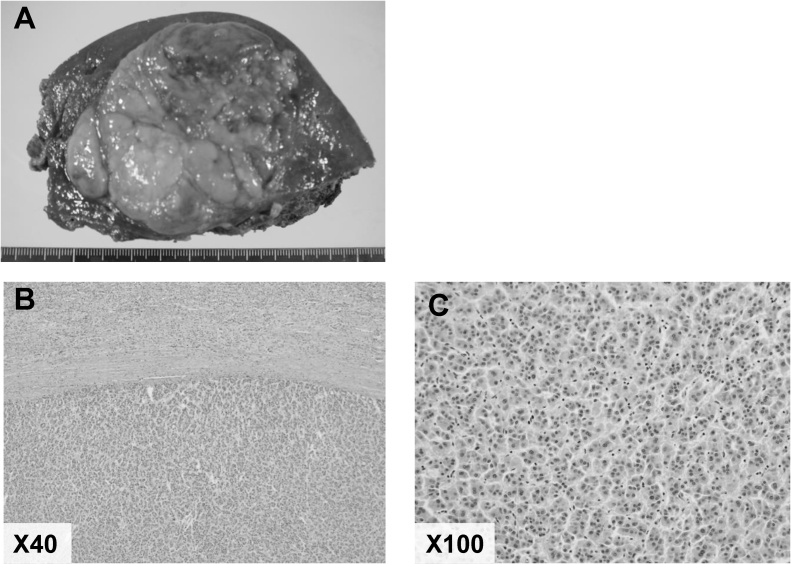
The resected specimens showed a confluent multinodular-type liver tumor (90 × 70 × 55 mm) (A). Pathological examination of the liver tumor revealed moderately differentiated HCC (B–C).

A 69-years-old man with chronic hepatitis B was admitted to our hospital for right subcostal pain and loss of appetite of four days duration. Laboratory data showed increased white blood cell of 12.7 × 10^3^/ μl, serum C-reactive protein of 20.88 mg/dl, total bilirubin of 1.8 mg/dl, aspartate aminotransferase of 56 IU/l, alanine aminotransferase of 72 U/l, alkaline phosphatase of 206 IU/l, and gamma glutamyl transferase of 93 IU/l. Abdominal computer tomography (CT) revealed wall thickening of the gallbladder with air density (Fig. 1A) and an 80-mm tumor with early enhancement in the segment 8 of the liver (Fig. 1B and C). With a diagnosis of emphysematous cholecystitis, percutaneous transhepatic gallbladder drainage (PTGBD) was performed. *Clostridium perfringens* was identified from the culture of bile. Gadoxetic acid-enhanced magnetic resonance imaging (MRI) revealed a liver tumor, which demonstrated hyper-vascularity in the arterial phase (Fig. 1D) and washout in the hepatocyte phase (Fig. 1E. Serum tumor markers were as followed; Carcinoembryonic antigen 1.8 ng/mL, alpha-fetoprotein 41 ng/mL, Protein Induced by Vitamin K Absence or antagonists-II (PIVKA-II) 1,120 mAU/ml. Therefore, we diagnose the liver tumor as hepatocellular carcinoma (HCC). The patients underwent extended anterior segmentectomy of the liver and cholecystectomy on 24 days after PTGBD. The gallbladder was necrotic and partially perforated, and peritoneal cavity was contaminated with yellow-white bile. A drain was placed in the right subphrenic space. The patient was given Flomoxef Sodium (FMOX) as perioperative antibiotic, which was sensitive for *Clostridium perfringens.* Pathological examination of the liver tumor revealed moderately differentiated HCC (Fig. 2A–C) with interface hepatitis, and gallbladder wall showed necrosis. *Escherichia coli* was cultured from the intraoperative bile.

**Fig. 3:**
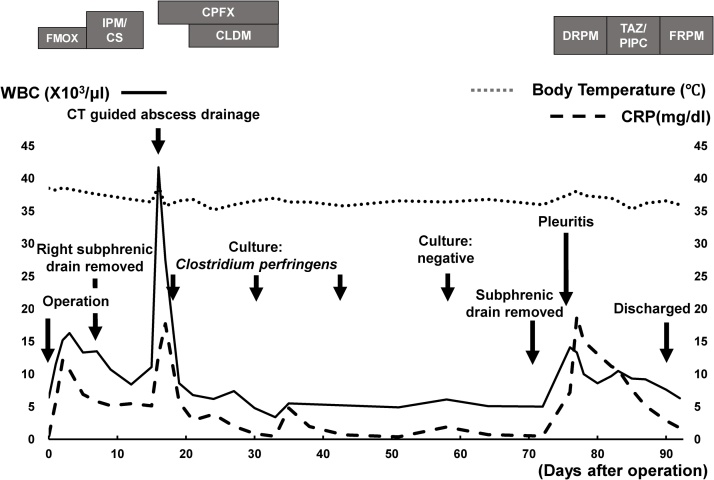
Clinical course of the patient, after operation. The right subphrenic drain was removed on postoperative day (POD) 5, but the patient developed fever on POD 15. CT-guided abscess drainage was performed for emphysematous subphrenic abscess on POD 16 and Ciprofloxacin and Clindamycin were given. *Clostridium perfringens* was cultured from the drainage fluid. The drain was removed on POD 75 after confirming negative culture of the drainage fluid. The patient was discharged on POD 95. Abbreviations: FMOX, Flomoxef Sodium; IPM/CS, Imipenem/Cilastatin; CPFX, Ciprofloxacin; CLDM, Clindamycin; DRPM, Doripenem; TAZ/PIPC, Tazobactam/Piperacillin hydrate; FRPM, Faropenem.

**Fig. 4:**
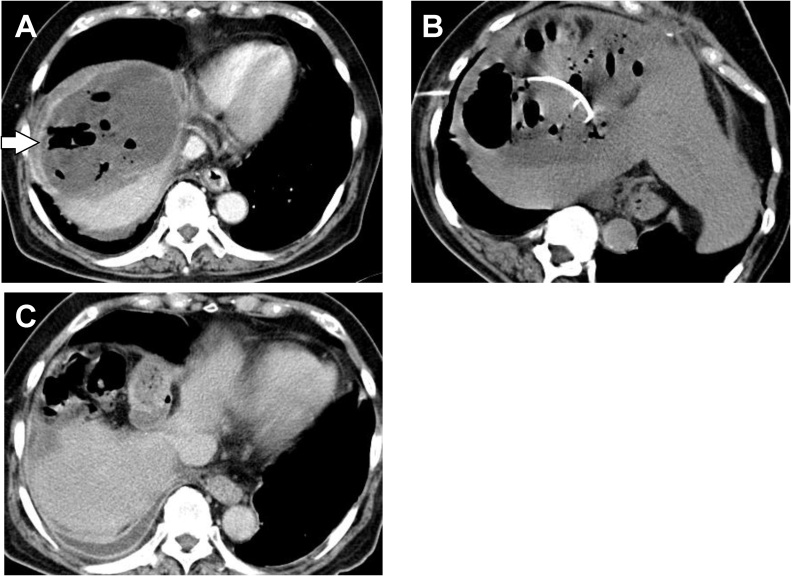
Computed tomography (CT) showed emphysematous subphrenic abscess on postoperative day POD 15 (A) and CT-guided percutaneous abscess drainage was performed on POD 16 (B). Subphrenic abscess disappeared on CT before the patient was discharged on POD 95 (C).

After the operation, the drain was removed on postoperative day (POD) 5 (Fig. 3). However, the patient developed fever on POD 15 and CT demonstrated the emphysematous subphrenic abscess (Fig. 4A), for which CT guided percutaneous abscess drainage was performed (Fig. 4B) and Ciprofloxacin and Clindamycin were started intravenously. *Clostridium perfringens* was cultured from the drainage fluid which was sensitive to Ciprofloxacin. Thereafter, the patient made a satisfactory recovery, and the drain was removed on POD 75 after confirming that the drainage fluid become negative. After confirming disappearance of subphrenic abscess on CT, the patient was discharged on POD 95 (Fig. 4C) and remains well with no evidence of tumor or abscess recurrence as of 8 years after resection.

## 3 Discussion

Subphrenic abscesses develop after surgery, trauma or intraperitoneal infection. Postoperative subphrenic abscess commonly complicate 1–3 weeks after biliary tract and gastric operations [3]. As for hepatic resection, subphrenic abscess has been attributed the necrosis of hepatic resection site tissue, large dead space, and contamination through drain in which the main pathogen of the abscess has been reported as gram-positive cocci (*Enterococcus, Staphylococcus aureus*) and gram-negative rods (*E. coli*) [4,5]. Surgical intervention with drainage of the abscess and antibiotics are important to treat subphrenic abscess [3,6].

*Clostridium perfringens* is gram-positive anaerobic bacilli which habitually reside in the gastrointestinal tract. *Clostridium perfringens* is reported to cause cholecystitis, liver abscess, intrauterine infection, and pyelonephritis with rapid progression [[7], [8], [9]], of which the biliary tree was most frequent site of infection [9]. Malignant tumor, diabetes mellitus, liver cirrhosis, and immunosuppressive conditions have been reported as risk factors for *Clostridium perfringens* infection [10,11]. *Clostridium perfringens* can cause a range of infections, from asymptomatic bacteremia to shock and death. Sepsis by *Clostridium perfringens* infection is characterized by disseminated intravascular coagulation (DIC), severe hemolysis, necrosis and multiple organ failure rapidly [9,10]. The mortality rate of patients with sepsis due to *Clostridium perfringens* has been reported to be approximately 70–80 % [7,12], with 30-day mortality rate of 27–44 % [7,11]. Antitoxin for alpha toxin (dry gas gangrenum equine antitoxin) has been used to treat sepsis by *Clostridium perfringens*. Early antibiotic and surgical treatment for the focus of infection such as cholecystectomy and drainage for abscess is associated with significant increase in the survival rate [7].

Postoperative intraperitoneal septic abscess caused by *Clostridium perfringens* is extremely rare, and has been reported as a complication after cholecystectomy, pancreatectomy and bile duct surgery [13,14]. To the best of our knowledge, this case is the first report of subphrenic abscess caused by *Clostridium perfringens* after hepatic resection. In the present case, although we performed peritoneal lavage thoroughly at operation and sensitive perioperative antibiotics were administered as perioperative treatment, *Clostridium perfringens* developed emphysematous subphrenic abscess on the large dead space of hepatic resection. The possible reasons are that the production of alpha-toxin may have been activated in the immunosuppressive condition after intraoperative red blood cell transfection following the excessive intraoperative blood loss, and in the anaerobic condition caused by necrotic tissue of hepatic resection site. Moreover, low hepatic functional reserve with chronic hepatitis B could be a risk factor of subphrenic abscess.

## 4 Conclusion

We herein reported a subphrenic abscess due to *Clostridium perfringens* after hepatic resection for hepatocellular carcinoma following emphysematous cholecystitis. When infection due to *Clostridium perfringens* is suspected, rapid recognition by Gram staining and surgical treatment are critical to improve outcomes.

## Sources of funding

There is no source of funding.

## Ethical approval

This study has been exempted by our institution.

## Consent

Written informed consent was obtained from the patients for publication of this case report and any accompanying images.

## Author contribution

RH carried out the acquisition of data and drafted the manuscript. KH, YK and SW were involved in the drafting of the manuscript. HS revised the manuscript. KY critically revised the manuscript. All authors read and approved the final manuscript.

## Registration of research studies

This is not ‘first-in-man study’.

## Guarantor

Ryoga Hamura.

## Provenance and peer review

Not commissioned, externally peer-reviewed.

## Declaration of Competing Interest

The authors declare that they have no competing interest.
